# Healthcare providers’ perspectives on integrating NCDs into primary healthcare in Thailand: a mixed method study

**DOI:** 10.1186/s12961-021-00791-1

**Published:** 2021-11-27

**Authors:** Titiporn Tuangratananon, Sataporn Julchoo, Mathudara Phaiyarom, Warisa Panichkriangkrai, Nareerut Pudpong, Walaiporn Patcharanarumol, Viroj Tangcharoensathien

**Affiliations:** 1 Department of Health, Ministry of Public Health, 88/22 Tiwanon Rd, Amphoe Mueang, Nonthaburi, 11000 Thailand; 2 International Health Policy Program, Ministry of Public Health, Nonthaburi, Thailand

**Keywords:** NCDs, Health promotion, Prevention, Control, Primary health care, Thailand

## Abstract

**Background:**

In response to an increased health burden from non-communicable diseases (NCDs), primary health care (PHC) is effective platform to support NCDs prevention and control. This study aims to assess Thailand’s PHC capacity in providing NCDs services, identify enabling factors and challenges and provide policy recommendations for improvement.

**Methods:**

This cross-sectional mixed-method study was conducted between October 2019 and May 2020. Two provinces, one rich and one poor, were randomly selected and then a city and rural district from each province were randomly selected. From these 4 sites in the 2 provinces, all 56 PHC centres responded to a self-administrative questionnaire survey on their capacities and practices related to NCDs. A total of 79 participants from Provincial and District Health Offices, provincial and district hospitals, and PHC centres who are involved with NCDs participated in focus group discussions or in-depth interviews.

**Results:**

Strong health infrastructure, competent staff (however not with increased workload), essential medicines and secured budget boost PHC capacity to address NCDs prevention, control, case management, referral and rehabilitation. Community engagement through village health volunteers improves NCDs awareness, supports enrolment in screening and raises adherence to interventions. Village health volunteers, the crucial link between the health system and the community, are key in supporting health promotion and NCDs prevention and control. Collaboration between provincial and district hospitals in providing resources and technical support enhance the capacity of PHC centres to provide NCDs services. However, inconsistent national policy directions and uncertainty related to key performance indicators hamper progress in NCDs management at the operational level. The dynamic of urbanization and socialization, especially living in obesogenic environments, is one of the greatest challenges for dealing with NCDs.

**Conclusion:**

PHC centres play a vital role in NCDs prevention and control. Adequate human and financial resources and policy guidance are required to improve PHC performance in managing NCDs. Implementing best buy measures at national level provides synergies for NCDS control at PHC level.

**Supplementary Information:**

The online version contains supplementary material available at 10.1186/s12961-021-00791-1.

## Background

Globally, non-communicable diseases (NCDs) contribute to 70% of total mortality each year [1]. Moreover, around 75% of NCDs deaths and 82% of 16 million annual premature NCDs deaths (age 30–70 years) are in low- and middle-income countries [1, 2].

Various global health forums reiterate and foster commitment to prevent and control NCDs. For example, an Independent high-level commission on NCDs in 2018 [3]; the global action plan for the prevention and control of NCDs 2013–2020 [4]; and the Montevideo Roadmap 2018–2030 [5]. SDG target 3.4 aims to reduce premature NCDs deaths by one third by 2030 [6].

In 2016 in Thailand, NCDs accounted for 74% of total mortality, with leading causes of cardiovascular diseases (23%), cancers (18%), chronic respiratory diseases (6%), and diabetes (4%). In 2016, there were 399 100 NCDs deaths, and 14% of the Thai population are at risk of premature death from NCDs [7].

The global action plan on NCDs calls upon strengthening primary health care (PHC) for effective prevention and control of NCDs [4], emphasizing early diagnosis, treatment and management of complications. Evidence shows positive health outcomes from integrating NCDs at PHC level [8, 9], and a reduction of hospital admissions [10]. Primary care has shown to play a vital role in managing diabetes and hypertension successfully in South India [11], and chronic obstructive pulmonary diseases in Nepal [12]. Bangladesh, with support from WHO, also uses PHC as a major channel for implementing the Package of Essential Noncommunicable (PEN) disease interventions for primary health care [13]. Thailand’s experience of implementing PHC for NCD prevention and control contributes to the current literature and can provide lessons for other low- and middle-income countries during their initial phase of NCD control.

### Defining PHC in Thailand

To support citizens’ first point of contact with the health service, Thailand’s PHC services are provided in district catchment areas of about 50 000 people through a network of 10–15 PHC centres and a district hospital of 30–120 beds [14]. This PHC network is called the “district health systems”. It provides a comprehensive range of services including health promotion, disease surveillance, home healthcare, out-patient services with supervision and support by medical doctors from district hospitals.

Three decades of PHC infrastructure development since the 1970s has led to full geographical coverage with PHC centres in all sub-districts and district hospitals nationwide [14]. In 2018, 9806 PHC centres in 7255 sub-districts provided a range of health services to an average of 5000 citizens in each sub-district catchment area [15, 16].

Since 2002, Thailand has achieved universal health coverage through financial risk protection schemes which offered a comprehensive benefit package (including the whole range of NCDs interventions) free at the point of service [14]. Box 1 describes PHC governance, function and financing.

Box 1 PHC governance, function and financing The Ministry of Public Health owns almost all sub-district health centres; only a small fraction, 51 out of 9806 PHC centres, are transferred to local government [46]. In provincial cities, PHC centres run by Ministry of Public Health are the major providers while municipalities have no role. They are more active in education provision, infrastructure, water and sanitation. MOPH also owns all district, provincial and regional hospitals nationwide.

Over 1 million village health volunteers nationwide play critical roles in supporting PHC including screening of diabetes (using blood strip test) and hypertension (using electronic blood pressure instrument) in the target populations. In some areas, the volunteers support early cataract detection and visual acuity in the elderly through the use of a Snellen chart. Beyond NCDs, the volunteers also support PHC staff during visits to home- and bed-bound chronic patients as well as outreach school health services such as promoting oral hygiene and Fluoride mouth wash, and creating awareness of seasonal diseases in the communities, such as Dengue and seasonal influenza. They played a critical role in observing and reporting deaths in poultry during the 2004 H5N1 epidemic [37], and the current COVID-19 pandemic [47].

PHC centres, served by nurses and public health officers, were categorised by the size of catchment population as small (population less than 3000), medium (3000–8000), and large (over 8000). District hospitals, are categorized into four levels by sizes and service capacity: First level 3 (F3) hospitals have 30 beds served by 1–2 general practitioners or family physicians; F2 (30–90 beds) and F1 (60–120 beds) have more physicians but no specialists; middle level 2 hospitals have more than 120 beds served by 3–5 generalists, and at least two specialists from each of the key specialities (medicines, obstetric gynaecology, paediatric, surgery, orthopaedics and anaesthesiologist).

PHC centres and district hospitals form a network of service providers called “district health systems”. The network is the contractor provider for outpatient services for UCS members nationwide. Similarly, at provincial level, the provincial hospital and PHC centres in the city also serve as contractor providers.

District health systems for outpatient services are financed by the National Health Security Office based on an annual capitation fee, for which UCS members are required to register with a district health system. The cost of referral to provincial hospitals for outpatient services is paid by the district health system. Inpatient services are financed by NHSO based on a diagnostic related group.

A study shows that the Chronic Diseases Clinic Model for diabetes and hypertension at the PHC level has significantly shifted the care of NCDs patients from hospitals to PHC centres. This minimizes congestion in hospitals while achieving good clinical outcomes [17]. Shifting the care of NCDs patients from district hospitals to sub-district PHC is widely practiced. Physicians also rotate to provide clinical services to NCDs patients at PHC centres where there are more caseloads for some days of the week. As the district health system is the contractor provider network for members of the universal coverage scheme, clinical support and supplies of medicine from district hospitals to PHC centres facilitate the effectiveness of NCDs control.

Literature reviews identify evidence gaps on how PHC centres integrate NCDs services into current service provision designs for acute, maternal and child health conditions, and on what capacities are required for the effective management of NCDs.

There are three rationales for this study. First, Thailand has invested in and reached full geographical coverage of PHC through its district health system operating since the 1970s. Second, universal health coverage has matured after two decades of implementation since 2002. Third, preventing and treating NCDs is a national priority, in response to NCDs presenting a high burden as measured by loss of Disability Adjusted Life Year. Hence there is a need to assess how PHC is able to address the challenges posed by NCDs. This study aims to assess PHC capacities in managing NCDs, identify enabling factors and challenges, and provide policy recommendations for improvement. Lessons from integrated NCDs management at PHC level, is useful for both national policy use and other low- and middle-income countries that are strengthening their PHC to address NCD challenges.

## Methods

This study was conducted between October 2019 and May 2020. Due to time and resource constraints, a national representative survey was not possible and the case study method [18] was applied, with no intention to generalise findings for all of Thailand.

This study applied mixed-methods, using qualitative and quantitative approaches. The quantitative self-administered survey assesses PHC capacity and major constraints in managing NCDs in different settings such as urban and rural and rich and poor provinces.

### Study sites

The study sites were selected based on provincial economic status and NCDs mortality rate. Firstly, gross provincial product per capita, a proxy of socioeconomic status, was utilised. High and low socioeconomic provinces were defined as the top ten richest and poorest provinces in Thailand. Secondly, the provincial-specific NCDs mortality rate from the Department of Disease Control was retrieved. The average NCDs mortality rate in Thailand in 2018 was 114.28 per 100 000 population (min: 64.56, max: 199.49) [19]. In these ten richest and ten poorest provinces, out of the provinces that had higher than national average NCDs mortality, one province was randomly selected. Saraburi province in the Central Region represented the highest socioeconomic province with a NCDs mortality rate of 146.42 per 100 000 population, whereas, Phrae province in the Northern Region represented the lowest socioeconomic province with an NCDs mortality rate of 176.33 per 100 000 population. Both provinces have higher than national average NCDs mortality rate.

One city and one rural district of the two provinces were randomly selected to participate in this study (see Table 1). In the four districts, all PHC centres including district hospitals (in rural area) or provincial hospitals (in city), district public health offices were invited and participated in this study.

**Table 1:** Summary of the study sites characteristics

	Gross provincial product per capita (2017) (THB)	Economic status rank	NCDs mortality rate 2018 (per 100 000 population)
Saraburi	330 750	10th out of 77	146.42
Phrae	67 057	69th out of 77	176.33
National average	228 398		114.28

Source: socioeconomic data from Office of the National Economic and Social Development Council [45] and NCDs mortality rate from Division of Non-Communicable Diseases, Ministry of Public Health [19]

### Questionnaire development

Guided by findings from the literature review, a self-administered questionnaire and interview guides were developed, tested and finalized. The questionnaire comprised three parts: facility characteristics, responsibilities, and availability of essential resources. The content of these questions were discussed and finalized at an international expert meeting. See detail in Additional file 1.

### Data collection

Quantitative data was collected through self-administered questionnaire surveys, with one questionnaire for each PHC centre. In total, 56 PHC centres (38 in Phrae and 18 in Saraburi provinces), belonging to the MOPH, responded fully to the survey, which was a 100% response rate.

Qualitative information was collected from face-to-face in-depth interviews or focus group discussions (FGD), depending on personal convenience and venue arrangement. The interviews used a semi-structured questionnaire tool, which was developed by the research team, validated by national experts, and piloted and finalized by the research team. A total of 79 participants were purposively chosen from healthcare professionals who are responsible for NCDs in the PHC facilities, district hospitals staff members who provide technical support for PHC and treatment of referral cases, and officers at District Health Offices and Provincial Health Offices. See Table 2.

**Table 2:** Number of key informants in two selected provinces

PHC category	Saraburi		Phrae		Total
	Urban area (*N*)	Rural area (*N*)	Urban area (*N*)	Rural area (*N*)	
PHC centres	10	8	25	13	56
District hospital	–	3	–	1	4
Provincial hospital	9	–	2	–	11
District Public Health Office	1	1	1	2	5
Provincial Public Health Office	1	–	2	–	3
Total participants	21	12	30	16	79

*PHC* Primary Health Care

A total of 15 FGDs were conducted on 6–8 January 2020 at Long and Meung district of Phrae province (PHC centres, district health office, provincial health office, Phrae hospital and Long hospital) and on 9 and 12 March 2020 at Saraburi City and Phra Phutthabat district (PHC centres, district health office, provincial health office, Saraburi and Phra Phutthabat hospital). No obvious difference on participant age was observed. Each interview lasted 40–60 min; it was audio-recorded after written consent, and transcribed for qualitative analysis. See detail in Additional file 2.

### Data analysis

Quantitative data were analyzed using STATA SE 14 and descriptive statistics including mean, standard deviation and percentage which describe size, distribution and profile of PHC were reported. The qualitative approach applied a combination of deductive and inductive approaches. Researchers started first with the deductive method and developed themes related to PHC capacity in responses to NCD challenges; these themes were then translated into a semi-structured interview questionnaire and interview guidelines. Researchers performed inductive analysis where new information emerged from the in-depth interviews after completion of the fieldwork and applied additional thematic analysis.

Key findings were triangulated according to the data source triangulation method. Factual data were validated and verified with references in relevant documents. For opinion and perception by key informants, the research team applied cross-validation with different key informants and field observations by the research team.

### Ethical approval

Ethical approval was granted by the Institute for Human Research Protection, Thailand (Reference: IHRP 096/2562). All data are kept anonymous and dissemination of data is for academic purpose without individual attribution.

## Results

In 2018, in Saraburi’s urban area, the population was 98 839, of which 2788 were 0–5 years and 16 728 over 60 years. The Universal Coverage Scheme (UCS) covered 62% of the population; 22% through Social Health Insurance (SHI) and 11% through the Civil Servant Medical Benefits Scheme (CSMBS). In Saraburi’s rural area the population was 46 632 of which 1902 were 0–5 years and 8955 over 60 years. The UCS covered 75% of the population; 18% through SHI and 7% through CSMBS. The percentage of adults age 35 years and above who were screened for hypertension was 41.3% in urban areas and 84.2% in rural areas [20], and for diabetes, 39% in urban areas and 82% in rural areas [21].

In 2018, in Phrae’s urban area, the population was 88 498 of which 3207 were 0–5 years and 26 211 over 60 years. The UCS covered over 70% of the population; 8% through SHI, and 16% through CSMBS. In Phrae’s rural area, the population was 30 915 of which 16 222 were 0–5 years, and 90 755 over 60 years. A vast majority of 90% were covered by UCS, 4% through SHI and 5% through CSMBS. The percentage of adults age 35 years and above who were screened for hypertension and diabetes was 96% in urban areas and 95% in rural areas [22]. Cardiovascular risk assessment was performed on 95% of adults over 30 in urban areas and 76% in rural areas [23].

Three thematic areas emerged from key findings from the questionnaire survey, FGDs, in-depth interviews and triangulation with literature reviews and other key informants.

### Theme 1: PHC foundation and enabling factors

Findings show that a strong foundation for PHC is the result of continued policy and financial support, improved management and human resources as described below.

**Fig. 1:**
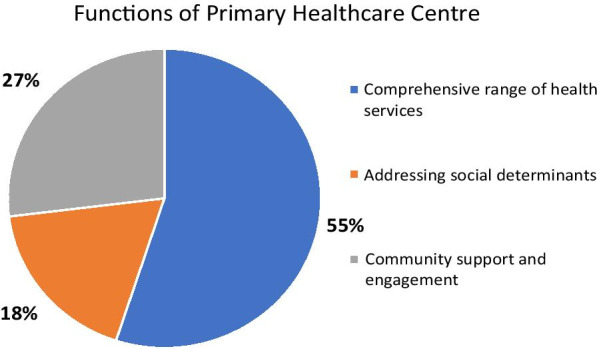
Three major functions of primary health centre, percentage of workload

**Fig. 2:**
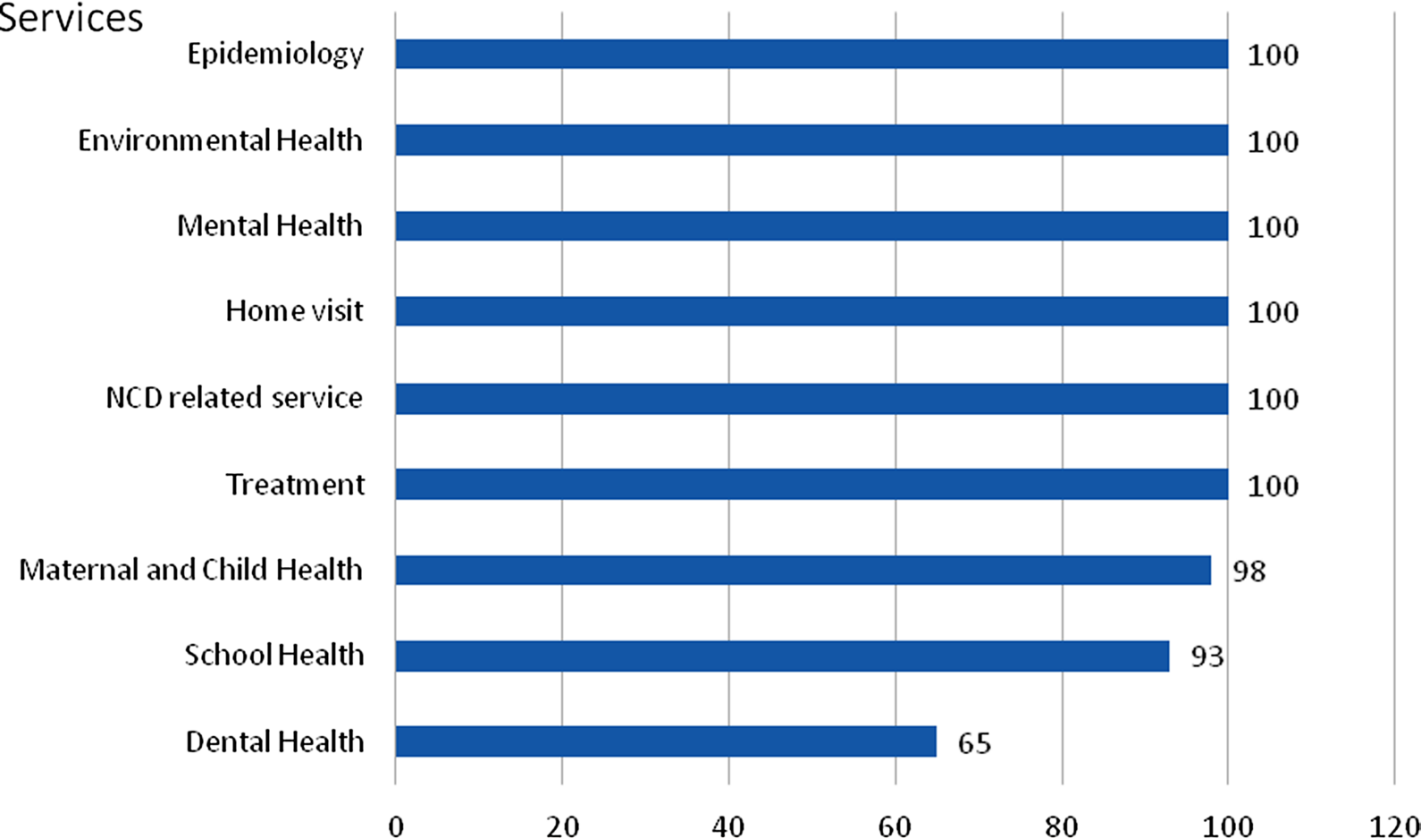
Services profiles provided by PHC centre, % of PHC centres. *NCDs* non-communicable disease, *PHC* Primary Health Care

1. PHC functions
  Self-administered questionnaire surveys found that PHC’s key function is to provide a comprehensive range of health services such as health promotion and disease prevention, treatment, and rehabilitation. This accounts for 55% of the total workload, of which NCDs take a major share. Around one third of the workload contributes to community engagement such as support to disabled, home-bound and bed-ridden patients. Inter-sectoral collaboration with local government units, which address the social determinants of health and empower citizens, accounts for 18% of PHC centres’ workload (see Fig. 1).
  The survey’s findings showed that PHC centres provide all services such as diseases surveillance, environment health, mental health, home visits, NCDs-related services and treatment. Dental health services were not provided by one third of 56 PHC centres due to the lack of dental personnel (Fig. 2).
2. PHC essential resources
  Self-administered questionnaire surveys also assessed essential resources for the functioning of PHC centres, include human resources and essential medicines.

#### Healthcare workers

This study categorizes PHC centres by the size of the catchment population: small (< 3000 population), medium (3000–8000) and large size (> 8000). The survey results revealed slight difference in numbers of staff by size, see Table 3.

**Table 3:** Human resources in three sizes of PHC centres

Size of PHC centre (catchment population)	Number of PHC centres	Average nurse	Average public health staff	Average dental nurse	Average total healthcare professional staff
Small (< 3000)					
Saraburi (urban)	5	1	2	0	3
Saraburi (rural)	4	1	2	0	3
Phrae (urban)	12	1	2	0	3
Phrae (rural)	10	1	2	0	3
Medium (3000–8000)					
Saraburi (urban)	4	2	2	1	5
Saraburi (rural)	4	2	2	1	4
Phrae (urban)	13	2	1	1	5
Phrae (rural)	3	2	2	1	5
Large (> 8000)					
Saraburi (urban)	1	3	2	1	8

*PHC* Primary Health Care

The total number of healthcare professionals (including nurses, public health officers and dental nurses) were three, five, and eight in small, medium and large size PHC centres, respectively. There is no difference in the number of health care workers between richer (Saraburi) and poorer (Phrae) provinces. The number of registered nurses, mostly post-graduate trained as Nurse Practitioners increased by the size of catchment population to accommodate more curative service workloads. In contrast, there are, on average, two public health officers who are 4-year trained regardless of size. A 4-year trained dental nurse and dental unit are only available in medium and large PHC centres.

Village health volunteers (VHVs) play a significant role in supporting PHC including screening of diabetes (blood strip test) and hypertension (electronic blood pressure instrument), supporting PHC staff during home visits and outreach school health services, and creating awareness of seasonal diseases, such as Dengue and influenza.

Monthly meetings between the VHVs and PHC centre staff are mandatory for refreshing VHV’s knowledge and getting updates on national, regional, and provincial health policies and guidelines. For example, to minimize exposure to COVID-19 infection at hospitals, NCDs cases were shifted to PHC centres and medicines were delivered by post. VHVs also supported local quarantine of individuals travelling from high infection areas or who had exposure to confirmed COVID-19 cases. (Note that L stands for key informants from low-income province, Phrae and H stands for key informants from high-income province Saraburi.)

*“We need to work hand-in-hand with local government units, community leaders and VHV. It is impossible to work successfully without support from other sectors and volunteers who know the community very well”* [L7]

#### Strengthen capacity of healthcare workers

In the past few years, the Ministry of Public Health (MOPH) earmarked budget for capacity building of PHC for NCDs case managers through the training of trainers. Although the budget was interrupted, some district hospitals initiated a mini-refresher course for their staff members to be NCDs case managers.

Within the province, all health facilities adhered to the clinical guidelines for case management and referral systems. Health professionals at PHC centres were well-qualified, and equity of health workforce density prevailed as the number of PHC staff was linked with the catchment population, regardless of the wealth of the province.

#### Availability of essential medicines

Essential medicines for NCDs case management suggested by World Health Organization (WHO) Package of Essential NCDs Interventions [24] is intended for use by physicians in PHC. See Table 4. Though most PHC centres in Thailand do not have a full-time physician, some part-time doctors from district hospitals provide NCDs services on-site in larger PHC centres. In this study, no PHC centre had full time physicians. Doctors provided part-time services in 43 of the total 56 PHC centres. The survey reports availability of essential medicines for diabetes, hypertension, and dyslipidaemia; and there were no stock outs of these essential medicines as they were managed by pharmacists in district hospitals. The following medicines were available in health centres at the following levels: Enalapril (98.1%), Simvastatin (98.1%), Metformin (98.1%), Aspirin (96.3%), Atenolol (96.3%), Amlodipine (94.4%), and Glucose injectable solution (92.6%). Only 27.8% of studied PHC centres reported availability of Glibenclamide as there are common renal side effects and must be prescribed by physicians who only come part time to some PHC centres. Certain items are not widely available in PHC centres, such as Isosorbide dinitrate (87.0%), Furosemide (85.2%), Thiazide diuretic (77.8%), Insulin (33.3%), and Spironolactone (20.4%) as these medicines required a physician’s prescription, according to key informants.

**Table 4:** Availability of essential medicines in PHC centres

Medicines	% available	Medicines	% available	Medicines	% available
Amoxicillin	100	**Furosemide**	85.2	**Spironolactone**	20.4
Paracetamol	100	Salbutamol	79.6	Diazepam	14.8
Ibuprofen	100	**Thiazide diuretic**	77.8	Codeine	11.1
**Enalapril**	98.1	Dextrose	72.2	Magnesium sulphate	7.4
**Simvastatin**	98.1	Erythromycin	64.8	Penicillin	7.4
**Metformin**	98.1	Sodium chloride infusion	53.7	Hydrocortisone	7.4
**Aspirin**	96.3	Senna	51.9	Beclomethasone	5.6
Oxygen	96.3	Epinephrine	38.9	Morphine	1.9
**Atenolol**	96.3	Glyceryl trinitrate	33.3	Heparin	0
**Amlodipine**	94.4	**Insulin**	33.3	Promethazine	0
**Glucose injectable solution**	92.6	**Glibenclamide**	27.8		
**Isosorbide dinitrate**	87.0	Prednisolone	24.1		

Bold text refers to medicines for management of NCDs

Since the launch of the Universal Coverage Scheme (UCS) in 2002, the National Health Security Office (NHSO) contracted the district health systems to fund PHC centres and district hospitals for outpatient services using a capitation payment method. All medicines and vaccines at PHC centres are procured and supplied monthly or bi-monthly by professional pharmacists in district hospitals. This ensures uninterrupted supplies of quality medical products at PHC centres. The capitation budget is adequate to provide services.

#### Additional financial resources

Since 2007, the NHSO has invested in the Local Health Promotion Fund (LHPF) at the sub-district level; it transfers 40 Baht per capita of catchment population in the sub-district, with an equal matching fund from the sub-district local government [25]. The Fund is an additional resource to respond to local health priorities. New NHSO guidelines in 2019 revised the scope of LHPF for the following priorities: (1) health services as prioritized by local community; (2) health promotion and disease control activities; (3) services for specific populations such as pre-school child development centres, the elderly and disabled people; (4) administrative costs of not more than 15% of the Fund to improve efficiency of Fund management; (5) control of disease outbreak and public health emergencies.

PHC plays a critical role in mobilizing resources generated by the Fund to support priority health problems in communities, with full engagement by citizens and local governments [26]. Most projects related to behavioural modifications and improved health literacy in relation to NCDs. Some projects supported target populations, such as monks, older people, disabled people and pregnant women. It should be noted that LHPF are implemented nation-wide with variation of outcome. In the study sites, key informants confirmed the significant role of LHPF in addressing local health challenges.

*“Paper media is outdated; we need secure funding for digital advertising media, we are able to mobilize resources from the Local Health Promotion Fund.”* [L1]

3. PHC service provision
  In addition to providing maternal and child health services such as antenatal care, family planning, immunization and child development clinics, PHC centres provided a wide range of screening and continued medication of well-controlled hypertension and diabetes, cervical cancer screening and follow-up for confirmed diagnosis of abnormal pap smear and treatment at provincial hospitals. Poorly controlled diabetic and hypertensive patients were referred to district hospitals. PHC centres also provided home visits for stroke, homebound and bedridden patients. All these services were mostly provided by postgraduate trained nurse practitioners. Some district hospitals with sufficient physicians, assigned physicians to work at large PHC centres.
  Local collaborations were initiated through multiple methods such as a Memorandum of understanding (MOU) between the Provincial or District Health Office and the local authorities (under the Ministry of Interior), or MOUs between the Provincial and District Health Office, District Health Coordinating Committee (DHCC), local nursing colleges, and private sectors. Some district or provincial hospitals had established NCDs committees, which comprise a multidisciplinary team.
4. Monitoring systems
  Clear national commitment is reflected by the key performance indicators (KPI) through a Quality Outcome Framework by NHSO to monitor service provision of specific interventions, in which all public healthcare facilities must participate. Incentives were given when a facility reaches the targets. At the same time, national KPI on health were set by MOPH for which the regional, provincial and district targets were then agreed and translated into annual work plans, implementation and regular monitoring. District hospitals and PHC centres were required to achieve these NCDs targets, with financial incentives from NHSO if targets are reached. Inspection of the quality of services was continuously monitored by NHSO while Social Security Office (SSO) and Comptroller Generals Department (CGD) were less active. Further the MOPH Inspector Generals also monitored target achievements in their supervisory visits.
  For example, in the last 5 years a target was set for 80% of diabetic and hypertensive individuals to be screened for Chronic Kidney Disease (CKD); 90% of the Thai population aged 35–74 were to be screened for Diabetes Mellitus (DM) through fasting blood sugar and at least 70% of DM type two aimed to achieved HbA1c < 7%. A cumulative 80% of women aged 30–60 were to be screened for cervical cancer [27].

### Theme 2: confusion and policy incoherence

Organizational disintegration, policy incoherence, lack of harmonization and dialogues among national policy makers and ineffective communication of decisions and guidelines to the implementation level, in particular PHC, all resulted in confusion at PHC centres. The provider–purchaser model is split, which means MOPH is the main provider while NHSO, CGD and SSO are the main purchasers. Each organization set their own rules and regulations relevant to their missions and legal mandates. Even within MOPH, Departments develop guidelines of which some details may be slightly different. This then sometimes creates policy incoherence across organizations. This theme mainly analyses the incoherence of financial resources, confusion of policy communication and its duplication, limited human resources and data management. This theme reflects the reality on the ground. Three sub-themes emerged which included policy incoherence from the national to the local level, insufficient or inappropriate budget management and human resources for health, and challenges of health information systems, which are all listed below.

#### Policy incoherence causes confusion at the local level

Multiple MOPH Departments have their own NCDs-related responsibilities; lack of harmonization leads to duplications of data requests and reporting from the local level. Incoherent policy was evident in three different age groups for hypertension screenings (over 25, 30 and 35 years) and responsibility lay in three different Departments. It should be noted that the Health Promotion Division of the Department of Health and the NCDs Division of Department of Diseases Control are both responsible for NCDs.

Sudden discontinuity of policy caused programme interruption; for example, school and community-based behavioural modification projects were terminated after a few years of implementation. This led to confusion at the PHC centres. There were also unclear NCDs job descriptions in the Health Promotion Unit and the NCDs Unit in the Provincial Health Office. Clear job descriptions are essential to ensure synergies and avoid duplication of efforts.

#### Insufficient or inappropriate budget management

Key informants raised the issue of conflict around budget allocation. The budget is transferred to the District Health Network (comprising a district hospital and a network of PHC centres), and usually the Director of the district hospital is the chair and the Head of District Health Office is the deputy chair of the network. Achieving targets of NCDs screening require major contributions by PHC centres, but they often lacked adequate budget, resulting in internal conflicts in the network. For example, PHC did not received adequate budget for screening HbA1C.

*“NCDs quality standard comprises several indicators, but often without adequate budget allocation to fulfil these mandates. As a result, our performance will be marked in the red zone due to budget shortfalls.”* [L7]

#### Limited human resources for health

Some NCDs services once provided by hospitals are increasingly shifted to PHC centres. There is increased demand for achieving KPIs. Yet incentives did not match the increased workload shouldered by the PHC centre staff. There was no dental assistant to increase scope such as annual oral check-ups for school children and a lack of physiotherapists limits rehabilitation services and multidisciplinary home visits.

*“The heart of PHC settings should be health promotion and prevention, but currently our effort focuses on the treatments of NCDs, though these are the immediate needs of the citizens.”* [L7]

#### Challenges of health information systems

Although the problem of data inaccuracy at the national Health Data Centre maintained by MOPH has improved, challenges remain. The variety of hospital software and information platforms led to the fragmentation of health data, despite efforts to harmonize and improve interoperability. Patient information was sometime entered and transferred manually, resulting in human errors.

Non-MOPH public healthcare facilities such as those under the Ministry of Interior and Ministry of Defence have their own data systems, software and platforms, which are yet to be incorporated into the National Health Data Centre for monitoring service coverage and health outcomes.

### Theme 3: dynamic social context: an emerging challenge

Urbanization and socialization rapidly transforms local contexts and bring on board challenges including lifestyles diseases. This is another concern raised by local PHC and echoed throughout the study.

#### Urbanisation and social influences

Rural PHC centres facilitate easy access to services and maintain good relationships with villagers and community leaders. Key informants confirm that patients prefer to seek health services from their local PHC centres rather than visiting over-crowded hospitals. Trust and interpersonal relationships within the community, built over years, influence people’s decisions to visit PHC centres.

*“People always choose the best option for themselves, therefore, easy access to PHC centres in their community are considered their best choice.”* [L7]

Greater challenges were echoed by key informants from urban PHC centres. Patients in urban areas, with various choices of private and public clinics and hospitals, often by-passed PHC centres. In addition, some private companies offered private insurance to employees who often used private hospital services. Coverage of NCDs screening and treatment outcomes by the private sector are unknown, as this information was not captured by the MOPH information system.

Urban populations live in obesogenic environments, having more access to fast food, sweetened beverages and inadequate physical activity than their rural counterparts. Energy-dense foods are key risks to obesity and NCDs. Key informants also voiced that health promotion around smoking, alcohol, and physical activity is less effective if the population is not interested. Addressing commercial determinants of tobacco, alcohol and unhealthy diets through implementing WHO best buys measures is important, but beyond the capacity of PHC workers [28].

#### Health literacy at the heart of NCDs prevention and control

Many key informants suggested promoting health literacy through mass or local media and that the MOPH should monitor and take legal actions against the promotion of false-claim products related to NCDs. Promoting healthy diets through schools and community-based interventions requires parallel reforms for conducive food environments.

*“We have been discussing ‘Health Literacy’ for years without applying it in context. We should apply these principles instead of repeating our talk.”* [L3]

*“We have performed so many services, but health behaviour is entirely on patients’ practices which closely link with their health literacy.”* [H3]

**Fig. 3:**
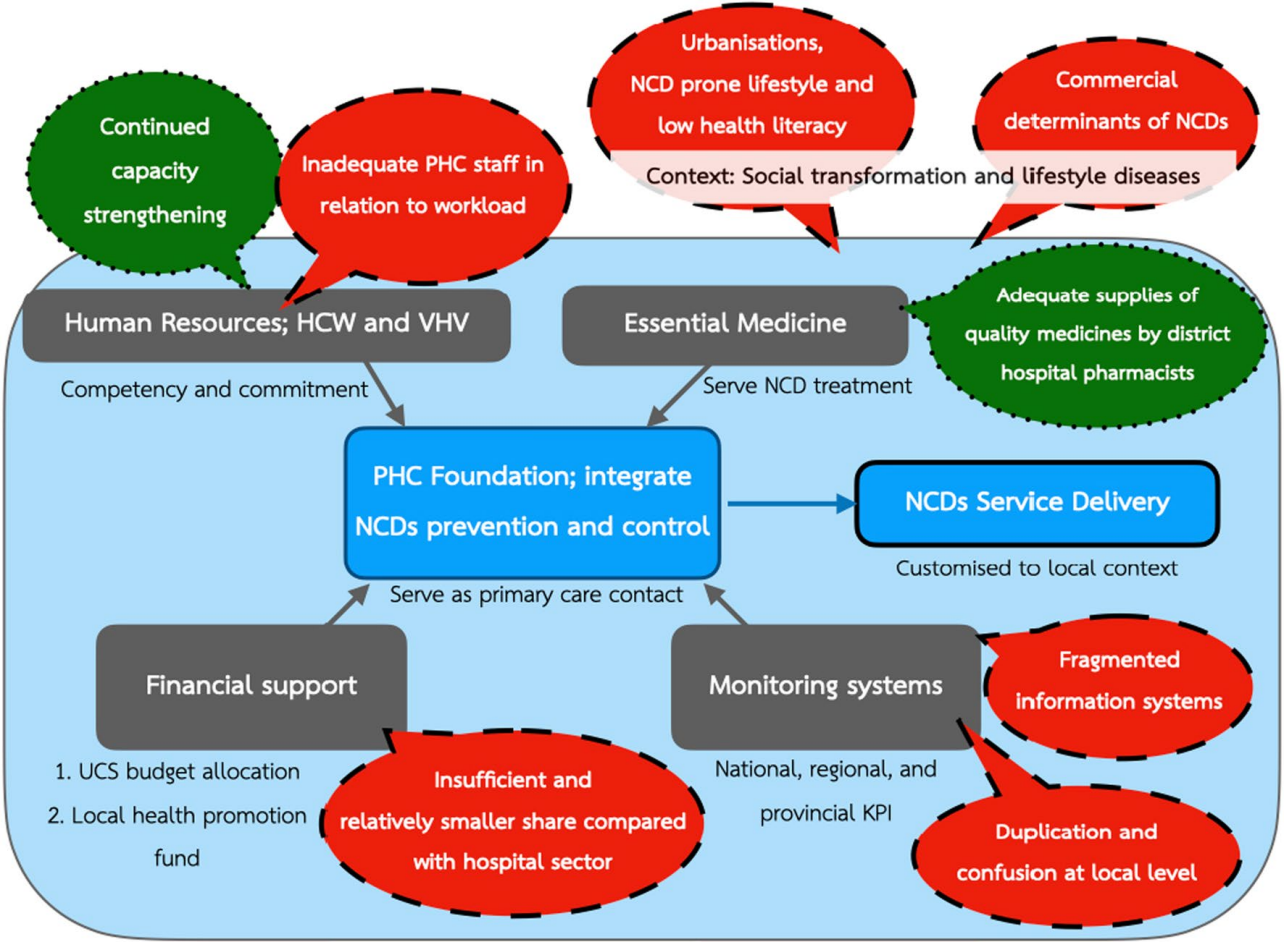
Thematic area on NCDs responses by PHC: enabling factors and challenges. *NCDs* non-communicable diseases, *PHC* Primary Healthcare Centre, *HCW* Healthcare Worker, *VHV* Village Health Volunteer, *UCS* Universal Coverage Scheme, *KPI* Key Performance Index. Dotted call outs refer to enabling factors and broken line call outs refer to challenges

Figure 3 depicts enabling factors that contribute to a strong PHC foundation, which in turn successfully integrate NCDs prevention and control at PHC level. Challenges are identified in the callout boxes.

**Fig. 4:**
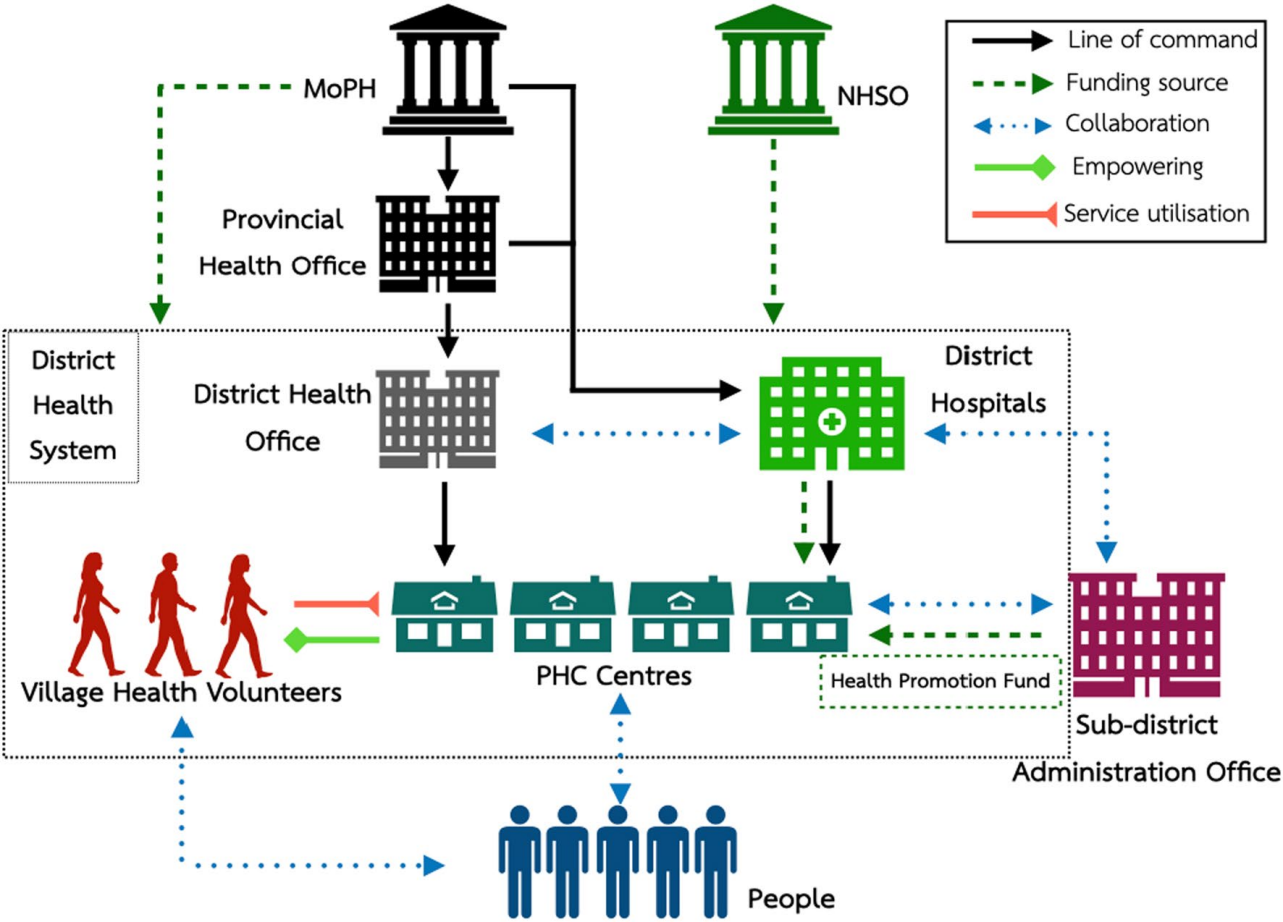
District health system: a key PHC platform for integrating NCDs prevention and control in Thailand. *PHC* Primary Healthcare Centre; *MoPH* Ministry of Public Health; *NHSO* National Health Security Office

Figure 4 depicts the district health system as a key PHC platform for integrating NCDs prevention and control in Thailand. Different key actors provide full support for the functioning of PHC including NCDs services.

## Discussion

Thailand achieved universal health coverage (UHC) in 2002 with the whole population covered by financial risk protection systems. The 9806 PHC centres in 7255 sub-districts nationwide provide the foundation for implementing UHC, as full geographical coverage contributes to equitable access to health services by all citizens [14, 29]. In past decades, PHC contributed to improved maternal and child health status, control of malaria and other infectious diseases. Today, PHC is also fit for purpose to respond to widespread NCDs. In 2017, government health spending was high at 15.03% of general government expenditure, and household out-of-pocket payments were 11.15% of health expenditure [30].

We estimated spending on PHC to be 40% of current health expenditure in 2017 and 38% in 2019 [31] Spending on PHC by Thailand is higher than in the OECD countries, which have an average of 14% of total health spending [32] but they are not comparable as the method of estimate differs. Data on PHC spending in low- and middle-income countries is however limited.

### Health workforce and financial resources: key determinants for functioning PHC

The success of PHC depends on the availability of qualified nurses and other health professionals in every PHC centre, often recruited from local communities for training and home-town placement upon graduation. Implementing rural health workforce retention policies such as local recruitment and hometown placements, and financial and non-financial incentives results in higher rural retention [33]. Qualified staff contribute to trust in quality PHC services by citizens [34]. Nurse practitioners are allowed to refill prescriptions in well-controlled NCDs patients; this increases access to care.

In-depth interviews with key informants showed difficulties in recruiting adequate numbers of dental nurses in small PHC centres due to limited posts and lack of career paths. The MOPH recognizes the problems but solutions have yet to be decided. A study shows 72.8% of dental nurses resigned due to frustration and heavy workloads [35]. However, it should be noted that this high rate of resignation may be due to other context specific areas.

Physicians confirmed diagnosis and choices of medication for NCD patients in district hospitals. Patients with well-controlled symptoms were referred to PHC centres. PHC staff members especially professional nurses monitored adherence to treatment, provided follow-up repeat medication and referred poorly controlled patients back to district hospitals. Medicines distributed at PHC centres were managed and supplied by pharmacists in district hospitals. Health literacy information such as risks from tobacco, alcohol, high fat and salty diets and physical inactivity was provided to NCD patients and community members by the PHC workers with assistance from the VHVs who closely engaged with the community and monitored their behaviour.

VHVs (1.054 million in 2020) are lay people in communities who are recruited to support PHC functions. Six 3-h modules are required for initial training of a new VHV [36] and a mandatory monthly one-hour refresher course is organized by PHC centres. Under the MOPH budget, VHVs are entitled to a monthly honorarium adjusted to 1000 Baht (US$ 35) in December 2018 from of 600 Baht (US$ 20) in 2008. During the 2004 H5N1 outbreak in Thailand, they also played critical roles to search and report sick and dead poultry in their villages [37].

When the COVID-19 pandemic peaked in March 2020, and the second half of 2021, VHVs played critical roles to monitor population mobility from high-risk areas and support home- and state-quarantine processes. Furthermore, VHVs are the key actors in identifying NCDs patients in the community, one of the priority targets for vaccinations, and in advising them to receive the COVID-19 vaccination, also ensuring that they have adequate NCDs medicines during the lockdown period. They also deliver NCDs medications to patients’ homes to prevent potential infection from spreading in the community and avoid patients coming into contact with large gatherings at PHC centres and district hospitals.

VHVs are given digital blood pressure devices and blood strips and are trained by PHC centres to monitor blood pressure and blood sugar in NCD patients and report back to PHC centres regularly. Further, VHVs had monthly meetings with and training by PHC staff members at PHC centres. See Form 1 for a sample of a diabetic and hypertension screening form conducted by a VHV. In 2020, MOPH announced the ‘three doctors per family’ policy [38], which include one VHV and one healthcare professional per 1250 population, and one family medicine doctor per 10 000 population. This was to ensure comprehensive care at the local level.
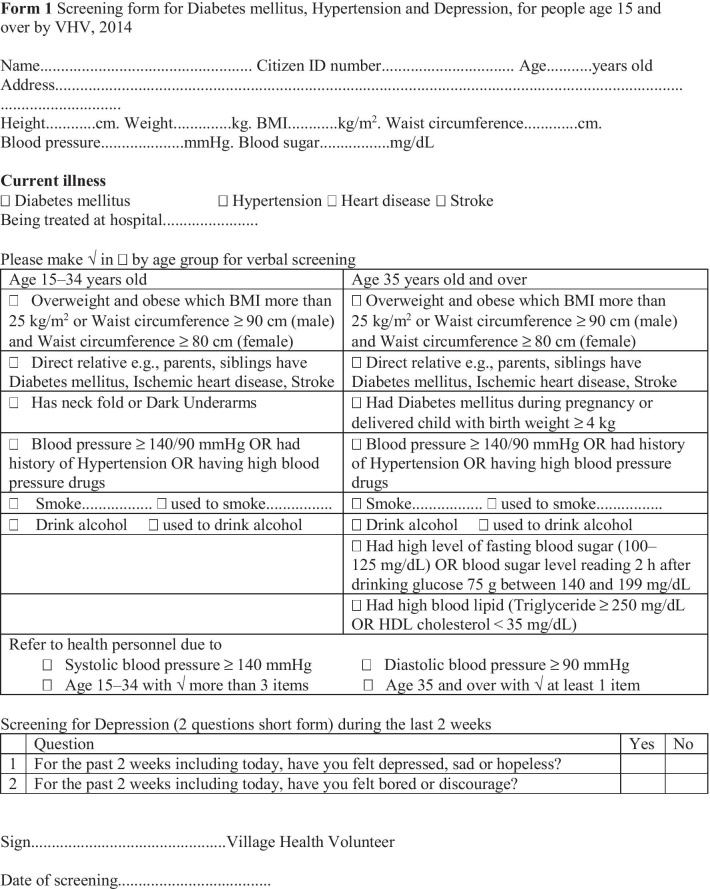


### PHC response to NCDs: a mixed outcome

The Astana Declaration defines three mandates for PHC. First, meet people’s health needs through providing a comprehensive range of health services throughout the life course; second, address the determinants of health through multi-sectoral actions; and third, empower individuals, families and communities to optimize their health, and support people as co-developers of health and social services [39].

Findings from this study confirm PHC can fulfil the first mandate on service provision and PHC centre health workers are fully trained for this purpose, including for NCDs. For example, 4 of 67 KPIs in 2020 were related to NCDs [40] and district, provincial and regional health authorities were required to report quarterly progress. Essential medicines for NCDs were available in more than 90% of PHC centres. Rotation of physicians to PHC centres boosts quality of care.

Although countries in the WHO South East Asia region had integrated NCD management at PHC level, most of them are small scale such as in Indonesia, India, Sir Lanka, Maldives, Myanmar, Nepal and Timor Leste. Bangladesh is at the initial phase of piloting. Only Bhutan and Thailand had implemented NCD management at PHC level nation-wide [41].

### PHC: challenges on multi-sectoral action

PHC is implemented with local government authorities through the DHCC creating spaces for multi-sectoral action. The DHCC supports law enforcement such as smoke-free public spaces, and legal sanction for violation of alcohol and tobacco sales to under-age children. PHC is also implemented through schools to promote healthy school lunches, and a ban on sugary sweeten beverages in schools [42].

Despite this, challenges remain on addressing the fundamental determinants of health. PHC is not designed and does not have capacity to allow for implementation of WHO best buy interventions [43] which can be most effectively achieved through national-level policy actions, effective law enforcement, such as increased taxes and prices, and the control of alcohol availability. One study shows successful results in advocating fizzy-drink-free schools by working closely with PHC centres, communities, local government and civil society organizations [44]. Scale up of these projects is underway.

### Study limitations

A few limitations exist. First, only two selected provinces have participated in this study, and findings cannot be generalized to the national level. The equal number of health workforce by size of catchment population in the two provinces, rich and poor, cannot be generalized either, and national level secondary data does not allow us to ascertain this finding. Second, the majority of participants were from PHC centres and in-depth interviews were dominated by PHC staff perspectives. Third, the COVID-19 situation in 2020, which enforced physical distancing and limited travel, did not allow interviews with representatives from the community, healthcare providers, Civil Society Organizations, VHVs, NHSO and the national NCDs manager.

Finally, the MOPH provides full scientific independence to researchers who are working in the MOPH. Hence, there is neither positive nor negative bias from the researchers’ side. However, field observation during in-depth interviews showed initial reluctance from key informants who were also MOPH civil servants to provide frank assessment. Upon ensuring confidentiality and rapport building, they then expressed the views frankly and constructively.

## Conclusion and recommendations

From the 1970s onwards it took three decades by successive governments to achieve full geographical coverage of PHC centres and district hospitals in the 1990s. An average of three to eight staff members in small, medium and large size PHC centres can accomplish the PHC mandate to provide comprehensive health services throughout the life course, including NCDs interventions for the sub-district catchment population. PHC staff members are trained to perform these functions well, and are supported by adequate supplies of essential medicines. Over 1 million VHVs play critical roles in bridging between PHC centres and communities and supporting diseases surveillance in times of public health emergencies.

Challenges remain to empower individuals and citizens to optimize their health, particularly in urban contexts. The PHC approach has limited capacity concerning multi-sectoral collaboration to address the social determinants of health as community-based interventions are not effective in implementing WHO best buy interventions. This study calls for improved coherence of policy and key performance indicators responsible by different departments in the MOPH prior to transcending to provincial, district and primary health care levels. This prevents unnecessary confusion and supports synchronized reporting by PHC centres.

To empower citizens and address social determinants through multi-sectoral action on NCDs, synergies and national-level support is needed for interventions such as tax and price policies on tobacco, alcohol and sweetened beverages, control of advertising, enforcing smoke free environments, and the availability and marketing of alcohol.

## Supplementary Information

**Additional file 1:** Self-administered questionnaires.

**Additional file 2:** Interview guidelines.

## Data Availability

All data collected and analysed in this study are included in this published article.

## Abbreviations

COVID-19: Novel Coronavirus
CGD: Comptroller Generals Department
CKD: Chronic Kidney Disease
CSMBS: Civil Servant Medical Benefits Scheme
DHCC: District Health Coordinating Committee
DM: Diabetes Mellitus
FGD: Focus group discussions
KPI: Key performance indicators
LHPF: Local Health Promotion Fund
MOPH: Ministry of Public Health
MOU: Memorandum of understanding
NCDs: Non-communicable diseases
NHSO: National Health Security Office
PHC: Primary health care
SHI: Social Health Insurance
SSO: Social Security Office
UCS: Universal Coverage Scheme
UHC: Universal health coverage
VHV: Village health volunteers
