# Supplementary material for: Healthcare providers’ perspectives on integrating NCDs into primary healthcare in Thailand: a mixed method study
Source: Health Res Policy Syst. 2021 Nov 27;19:139. doi: 10.1186/s12961-021-00791-1 (PMC8626719; doi:10.1186/s12961-021-00791-1)
Supplement: Supplementary file 1 — Additional file 1: Self-administered questionnaires. [file 12961_2021_791_MOESM1_ESM.docx]

**Additional file 1**

Self-administrated questionnaires

**Provisions of NCDS Services: Health Promotion,**

**Prevention and Control at Primary Health Care Questionnaire**

_____________________________________________________________________________

**Part 1 Healthcare facilities characteristics**

1.1 Healthcare facilities

🞏 Primary healthcare centre

🞏.Hospital level........(according to Ministry of Public Health category)

1.2 Line of command

🞏 Ministry of Public Health 🞏 Other

1.3 Population catchment area .....................

1.4 Total staff number ....... including;

| Physician ....... | Nurse ....... | Dentist ...... |
| --- | --- | --- |
| Dentist assistance ....... | Pharmacist ....... | Pharmacist assistance...... |
| Public health officer ...... | Nurse assistance....... | Physical therapist ....... |
| Traditional Thai physician ....... | Nutritionist.... | Social worker ....... |

**Part 2 Healthcare facilities responsibilities**

2.1 Describe three main functions of PHC and detail functions on provision of services by percent of total staff time.

| Main functions | % of total staff time  Facility type… |
| --- | --- |
| I. Provision of comprehensive range of promotive, protective, preventive, curative, rehabilitative, and palliative care throughout the life course. | A% |
| II. Addressing broader determinants of health through multi-sectoral actions | B% |
| III. Empowering individuals, families and communities to optimize their health, support people as co-developers of health and social services | C% |
| Total | 100% |

2.2 The provision of services in each PHC facility that should be asked can be seen the table below.

| Function provided at PHC facility | Yes/No | Yes/No |
| --- | --- | --- |
| - Maternal and child health services, such as antenatal care, delivery, family planning, post-natal care, immunization |  |  |
| - Basic curative services |  |  |
| - NCDS services including screening and case detection, treatment of NCDS conditions, follow up and referral to hospitals |  |  |
| - Dental health |  |  |
| - Mental health |  |  |
| - School health |  |  |
| - Basic sanitation and environmental health |  |  |
| - Surveillance of infectious diseases |  |  |
| - Home visit for chronic bed ridden patients and palliative care |  |  |

**Part 3 Essential medicines and medical devices in primary healthcare facilities**

Availability of essential medicines available for PHC facilities with Physicians

| **Essential medicines recommended**  **by PEN (PHC with Physician)** | **YES** | **NO** | **Essential medicines recommended**  **by PEN (PHC with Physician)** | **YES** | **NO** |
| --- | --- | --- | --- | --- | --- |
| Thiazide diuretic |  |  | Ibuprofen |  |  |
| Calcium channel blocker (amlodipine) |  |  | Codeine |  |  |
| Beta-blocker (atenolol) |  |  | Morphine |  |  |
| Angiotensin inhibitor (enalapril) |  |  | Penicillin |  |  |
| Statin (simvastatin) |  |  | Erythromycin |  |  |
| Insulin |  |  | Amoxicillin |  |  |
| Metformin |  |  | Hydrocortisone |  |  |
| Glibenclamide |  |  | Epinephrine |  |  |
| Isosorbide dinitrate |  |  | Heparin |  |  |
| Glyceryl trinitrate |  |  | Diazepam |  |  |
| Furosemide |  |  | Magnesium sulphate |  |  |
| Spironolactone |  |  | Promethazine |  |  |
| Salbutamol |  |  | Senna |  |  |
| Prednisolone |  |  | Dextrose infusion |  |  |
| Beclometasone |  |  | Glucose injectable solution |  |  |
| Aspirin |  |  | Sodium chloride infusion |  |  |
| Paracetamol |  |  | Oxygen |  |  |

Thank you for your kind cooperation in completing this questionnaire
