# Supplementary material for: Healthcare providers’ perspectives on integrating NCDs into primary healthcare in Thailand: a mixed method study
Source: Health Res Policy Syst. 2021 Nov 27;19:139. doi: 10.1186/s12961-021-00791-1 (PMC8626719; doi:10.1186/s12961-021-00791-1)
Supplement: Supplementary file 2 — Additional file 2: Interview guidelines. [file 12961_2021_791_MOESM2_ESM.docx]

**Additional file 2**

Interview guidelines

1. PHC health workforce, competency and capacity building
   - Describe capacity building / strengthening of PHC staffs in each year during 2016-2018
   - Specific skills include health promotion, prevention and control of NCDs. The content may include screening, detection, risk assessment, pharmacological and non-pharmacological interventions, capacity to use and interpret cardio-vascular risk score and life style modification, motivation interviewing skill, clinical management, prevention of secondary and tertiary complications such as renal failure, diabetic foot, diabetic retinopathy.
   - What models of training are used, such as “training of trainers”, so that cascade large scale training can be achieved?
   - How many of them were trained?
2. Assess the current competency and competency gaps of the health workforce as a team in providing NCDS services on-site according to following competencies


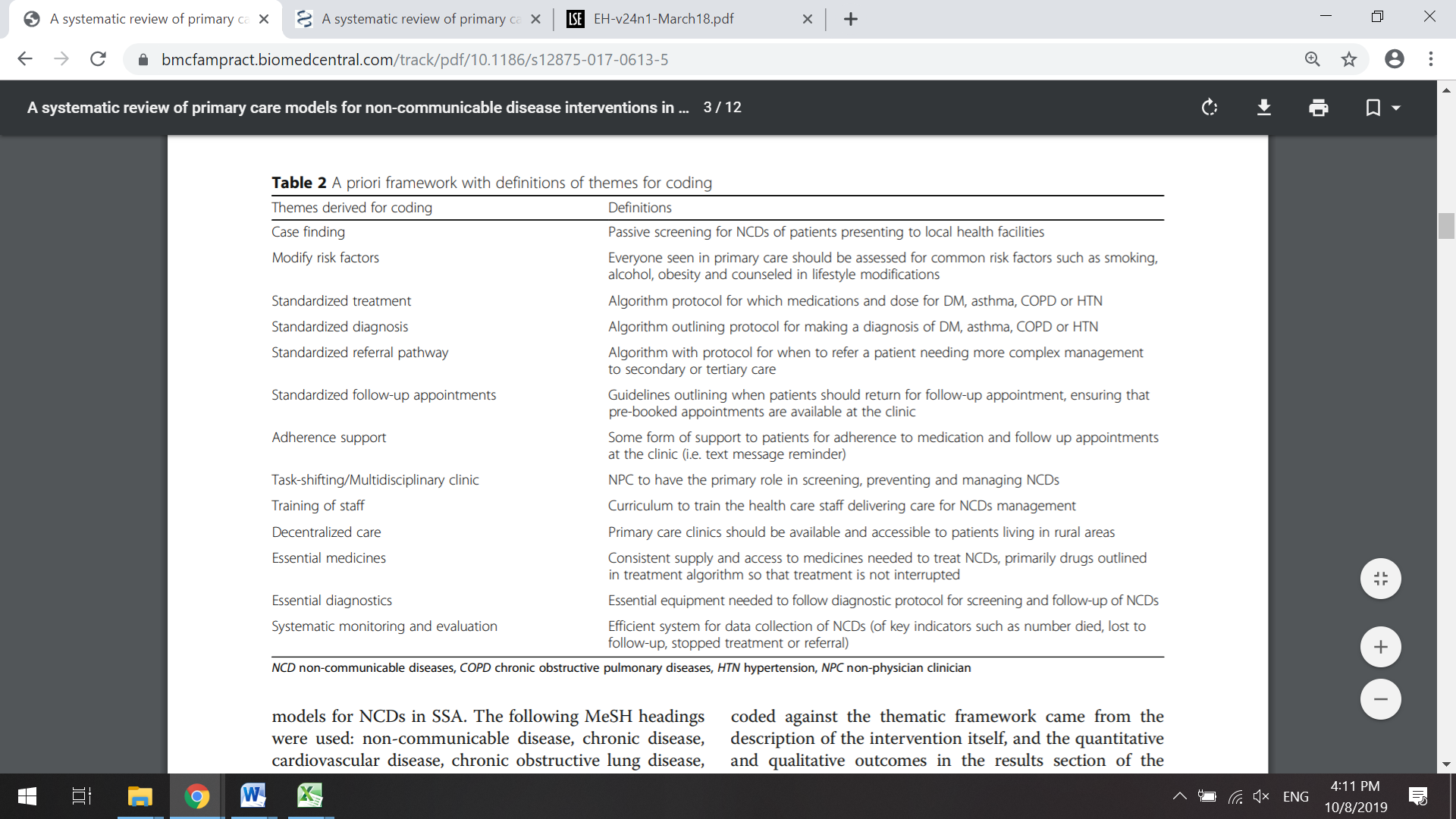


Source: A systematic review of primary care models for non-communicable disease interventions in Sub-Saharan Africa

Jennifer Kane, Megan Landes, Christopher Carroll, Amy Nolen1 and Sumeet Sodhi

Kane et al. BMC Family Practice (2017) 18:46

1. Function of primary healthcare facilities
2. Patient pathways and financial access to care

Describe the processes/pathways for which of population are screened and identified as no risk, high risk, or having NCDS conditions, and also complications

- Identify people at risk of NCDs and those with NCDs, with and without complications
- Laboratory confirmation diagnosis,
- Case management and follow-up;
- Pharmacologic and non-pharmacologic interventions;
- Support for adherence and modification of unhealthy behaviour, minimize risk, and self-care;
- Treatment guideline and management of complications such as stroke, myocardial infarction, end stage renal failure, end of life care
- Referral procedures;

1. Describe three main functions of PHC and detail functions on provision of services by per cent of total staff time of 100%, and describe the rationale of the following job allocation.

I. Provision of comprehensive range of promotive, protective, preventive, curative, rehabilitative, and palliative care throughout the life course.

II. Addressing broader determinants of health through multi-sectoral actions

III. Empowering individuals, families and communities to optimize their health, support people as co-developers of health and social services

1. Does your PHC facility provide the services indicated below? Please also explain the absent of services, if applicable.

• Maternal and child health services, such as antenatal care, delivery, family planning, post-natal care, immunization

• Basic curative services

• NCDS services including screening and case detection, treatment of NCDS conditions, follow up and referral to hospitals

• Dental health

• Mental health

• School health

• Basic sanitation and environmental health

• Surveillance of infectious diseases

• Home visit for chronic bed ridden patients and palliative care

1. Essential medicines and medical devices
   - Does your PHC receive sufficient medicines and medical devices? In case of insufficiency, what are the solutions provided?
   - Where does your PHC receive budget or actual product of medicines and medical devices?
2. Patient care and financial mobility
   - Identify and describe the barriers of access to NCDs services: diagnostic, medicine and high cost medicines for treatment of complications. How much on average the uninsured patients have to pay for diagnosis, and medications for outpatient and inpatients at hospitals as well as transportation. How much on average the insured patients have to pay for NCDS services as well as transportation cost?
   - What is the average, range of annual follow up visits for outpatient at PHC for DM and Hypertension? Average transport cost and time cost for follow up visit at PHC shouldered by patients?
   - Describe which Information, Education and Communication medias are made available at PHC in local language which empower NCDS patients on risk factors, life style modification and adherence to pharmacological and non-pharmacological interventions.
3. Patient referral systems for secondary hospitals
   - Please describe what supports (technical, supervision, training and capacity building, case management, referral, supplies of essential medicines and medical technologies) PHC received from secondary level hospitals for management of NCDS patients.
   - Identify good practice or barriers for these supports
   - Describe how patient access to PHC services
   - Describe additional services provided for NCDS patients in order to increase treatment efficiency, access to medicine, drug compliance, or home visit.
   - Is there any circumstance for patient who may pay out-of-pocket for treatment?
   - Describe the referral systems with private sectors (private clinics, pharmacy stores, private laboratories), civil society, and non-governmental organisation.
4. Monitoring and Evaluation and Information systems
   - Did the MOH establish key performance indicators on NCDS for which PHC need to comply with? If yes, what level of Key Performance Index this PHC had achieved?
   - Describe health management information system for registering, treatment, follow up, and monitoring such as level of Haemoglobin A1c in diabetes patients, selection of monitoring and evaluation indicators such as percent DM patient with well-control blood sugar, percent hypertensive patients with well-control blood pressure.
   - Describe the data flow of your PHC, where is the terminal data analysis centre (if applicable)?
   - Does your PHC perform indicator analysis such as well-control diabetes or hypertension data?
5. Provision of cost-effective NCDS interventions

Describe what tools below, are made available and are used at PHC, in local language and tailor made for health systems context

| **Tools** | **AVAILABLE** | | **USED by professionals** | |
| --- | --- | --- | --- | --- |
|  | **YES** | **NO** | **YES** | **NO** |
| 1. WHO/ISH risk prediction charts* |  |  |  |  |
| 1. Evidence based clinical protocols |  |  |  |  |
| 1. Flow charts with referral criteria |  |  |  |  |
| 1. Patient clinical record |  |  |  |  |
| 1. Medical information register |  |  |  |  |
| 1. AUDIT tools (C and standard) ** |  |  |  |  |

Note: * the objective of WHO/ISH risk prediction charts is to quantify the 10-year cardiovascular risk of a fatal or non-fatal major cardiovascular event (myocardial infarction or stroke), according to age, sex, blood pressure, smoking status, total blood cholesterol and presence or absence of diabetes mellitus?
(See Annex 2 on WHO/ISH risk predictor charts.)

** AUDIT tool <https://www.integration.samhsa.gov/images/res/tool_auditc.pdf>

- Did WHO /ISH risk prediction charts were translated into local language; publicly available, used for training health workers ^6^?
- Can health workers interpret and classified high risk individual (maroon and red colour), medium risk (orange and yellow), and low risk (green) for heart attack or stroke in the next ten years?
- Did immediate treatment actions are given for high risk individuals? Did counselling such as behaviour modification, tobacco cessation, adherence to treatment of diabetic, hypertension, advocate more physical activities, low salt, low fat diet are provided after the diagnosis?
- Did AUDIT Tools were translated into local language; publicly available, used for training health workers?

1. Public health solutions: provision of NCDS services through a lifespan approach.

To what extent these interventions are implemented by PHC? What enabling factors or barriers providing NCDS services?

Infancy:

- Exclusive breast feeding for 6 months
- Nutritionally adequate and safe complementary feeding starting from the age of 6 month with continued breastfeeding up to 2 years of age or beyond.

Childhood and adolescence:

- Improve life skills education;
- Promote physical activity in school and society;
- Safe and healthy foods in schools;
- Restrict marketing of and access to food products high in salt/sugar/unhealthy fats;
- Institute tobacco and alcohol controls.

Adulthood:

- improve maternal nutrition;
- implement tobacco prevention and cessation programmes;
- improve availability and affordability of food;
- encourage physical activity (worksites, urban design);
- provide access to effective prevention and care of risks and diseases.

1. To what extent these services were provided at PHC? What enabling factors or barriers in providing these NCDS services?

**Primary prevention**

1. Tobacco cessation (level 1)

2. Regular physical activity 30 minutes a day (level 1)

3. Reduced intake of salt <5 g per day (level 1)

4. Fruits and vegetables at least 400g per day (Level 2)

5. Aspirin, statins and anti-hypertensive medication for people with 10-year cardiovascular risk >30% (Level 1)

6. Anti-hypertensive medication for people with blood pressure ≥160/100 mmHg

7. Anti-hypertensive medication for people with persistent blood pressure ≥140/90 mmHg and 10-year cardiovascular risk >20% unable to lower blood pressure through life style measures (Level 1)

**Secondary prevention**

1. Ensure compliance of aspirin, antihypertensive, statin (Level 1)

2. Daily insulin injections in Type 1 Diabetes (Level 1)

Type 2 diabetes:

1. Oral hypoglycaemic agents for type 2 diabetes, if glycemic targets are not achieved with modification of diet, maintenance of a healthy body weight and regular physical activity (Level 1)

2. Metformin as initial drug in overweight patients (Level 1) and non-overweight (Level 4).

3. Other classes of antihyperglycaemic agents, added to metformin if glycaemic targets are not met (Level 3)

4. Reduction of cardiovascular risk for those with diabetes and 10-year cardiovascular risk >20% with aspirin, angiotensin converting enzyme inhibitor and statins (Level 1)

**Tertiary prevention in Diabetes:**

1. Optimal glycaemic control in people with type 1 or type 2 diabetes (Level 1)

2. Regular (3-6 months) visual inspection and examination of patients’ feet by trained personnel for the detection of risk factors for ulceration (assessment of foot sensation, palpation of foot pulses inspection for any foot deformity, inspection of footwear) and referral as appropriate

3. Angiotensin converting enzyme inhibitor for persistent albuminuria (Level 1)

4. Referral for screening and evaluation for laser treatment for diabetic retinopathy (Level 1)

14. Please describe gaps or challenges and proposed solutions on NCDS services and general PHC services from practitioners’ views.
